# Smallpox during Pregnancy and Maternal Outcomes

**DOI:** 10.3201/eid1207.051531

**Published:** 2006-07

**Authors:** Hiroshi Nishiura

**Affiliations:** * University of Tübingen, Tübingen, Germany;; † Hiroshima University, Hiroshima, Japan

**Keywords:** Epidemiology, Smallpox, Pregnancy outcomes, Case fatality, Miscarriage, Premature birth, Vaccination

## Abstract

A historical study evaluated maternal outcomes in pregnancy complicated by smallpox. The overall case fatality was estimated to be 34.3% (95% confidence interval [CI] 31.4–37.1), and the proportion of miscarriage or premature birth was estimated to be 39.9% (95% CI 36.5–43.2). Vaccination before pregnancy reduced the risk for death.

Pregnant women are at special risk for complications of smallpox vaccination (*1*); therefore, vaccination is not recommended for pregnant women in the absence of a reemergence of smallpox (*2*). Smallpox in pregnancy is believed to be more severe than in nonpregnant women or adult men (*3*), but this consensus is based on a limited number of studies conducted during the mid-20th century (*4**–**6*). This article examines the outcomes of pregnancy complicated by smallpox in historical records from the 19th and 20th centuries.

## The Study

Since most large outbreaks were documented before the mid-20th century, I collected and reviewed the literature dating back to the 19th century. Technical details of the literature review are provided in online Appendix 1. All selected publications were retrospective studies based on epidemiologic observations of outbreaks that reported case fatalities, miscarriages, or premature births. Because vaccination or advances in obstetrics over time could bias these outcomes, these factors were abstracted from each publication and considered separately, when possible. Outcomes were then stratified by gestation period at onset of smallpox (by trimester), clinical classification of smallpox, and vaccination history. Case fatalities were compared between pregnant and nonpregnant patients. Except in Rao's work in Madras (*4*), miscarriage and premature birth were not separated, so they are described together.

Nineteen outbreaks were identified from historical records (*4**,**7**–**20*), and of these, 16 allowed estimates to be made of case fatality, and 15 allowed estimates of the proportion of miscarriage or premature birth. Of 1,074 pregnant patients, 368 died; and of 830 pregnant patients, 331 miscarried or gave birth prematurely (Figure). Since these articles are from many years ago, the proportion of cases that were undetected or unreported cannot be determined nor can the length of time since vaccination in persons who were vaccinated. Descriptions of excluded literature are given in online Appendix 1; individual case records were provided in 3 outbreaks and are included in online Appendix 2.

Figure, panel A, shows the distribution of estimated case fatalities for each outbreak with the corresponding 95% confidence intervals (CIs). Case fatalities varied widely among outbreaks. The earliest outbreak in 1830 (before compulsory vaccination) yielded the highest estimate (81.5%), while the 1913 outbreak in Australia had the lowest (4.3%). The overall crude case fatality was estimated to be 34.3% (95% CI 31.4–37.1). Case fatality, stratified by gestational age at onset of smallpox, is presented in Table 1; only 4 studies enabled stratification by gestational age. Case fatality was highest during the third trimester, except in Queirel's study, which included few cases (*18*). Case fatality, stratified by the clinical classification of smallpox, is shown in online Appendix 2. All patients with hemorrhagic cases died, but all patients without a rash (variola sine eruptione, VSE) survived.

**Table 1:** Case fatality among pregnant women with smallpox by gestational age, according to data from 19th- and early 20th-century outbreaks*

Reference	Gestational age <3 mo		Gestational age 4–6 mo		Gestational age 7–9 mo	
	D/C	CF (95% CI)	D/C	CF (95% CI)	D/C	CF (95% CI)
Meyer (*9*), 1868–1872	3/33	9.0 (0.0–18.9)	11/33	33.3 (17.2–49.4)	8/10	80.0 (55.2–100.0)
Welch (*10*), 1878	4/12	33.3 (6.7–60.0)	4/22	18.2 (2.1–34.3)	6/12	50.0 (21.7–78.3)
Queirel (*18*), 1906	2/4	50.0 (1.0–99.0)	7/10	14.5 (41.6–98.4)	1/5	17.9 (0.0–55.1)
Rao (*5*), 1959–1962	7/21	33.3 (13.2–53.5)	16/65	24.6 (14.1–35.1)	34/94	36.2 (26.5–45.9)
Total	16/70	22.9 (2.3–43.4)	38/130	29.2 (14.8–43.7)	49/121	40.5 (26.8–54.2)

*D/C, smallpox deaths/cases; CF, case fatality; CI, confidence interval.

Case fatalities among pregnant and nonpregnant patients are compared in Appendix 2. Case fatality was not significantly higher in pregnant patients in the Rotterdam outbreak (p = 0.33), where many VSE cases apparently occurred. The risks for a fatal outcome among pregnant patients in Berlin and Madras were 2.5× and 4.2× higher than among nonpregnant patients (p<0.01 for each). I also compared vaccinated and unvaccinated pregnant patients, showing that the risk for death was significantly higher among unvaccinated women in these 3 outbreaks (7/7 vs. 7/39, p<0.01; 2/2 vs. 10/78, p = 0.02; and 9/12 vs. 17/82, p<0.01, respectively).

Crude proportions of miscarriage and premature birth, with 95% CI, are given in the Figure, panel B. The overall crude proportion of miscarriage or premature birth is estimated to be 39.9% (95% CI 36.5–43.2). Five outbreaks allowed stratification by gestational age at onset of smallpox (Table 2). The overall proportion of premature birth was highest during the last trimester of pregnancy, but no clear pattern was seen with regard to the frequency of miscarriage or premature birth. The proportion of miscarriage and premature birth, stratified by severity of smallpox, is shown in online Appendix 2. All hemorrhagic cases resulted in either miscarriage or premature birth before the mother's death. Even mild cases, those classified as discrete or VSE, tended to result in miscarriage or premature birth. Only the 1878 outbreak in Philadelphia (*10*) allowed a comparison between vaccinated and unvaccinated pregnant patients. Twenty-two of 39 vaccinated and 5 of 7 unvaccinated patients miscarried or delivered prematurely (p = 0.68).

**Table 2:** Miscarriage or premature birth among pregnant women with smallpox by gestational age, according to data from 19th- and early 20th-century outbreaks*

Reference	Gestational age <3 mo		Gestational age 4–6 mo		Gestational age 7–9 mo	
	L/C	PL (95% CI)	L/C	PL (95% CI)	L/C	PL (95% CI)
Meyer (*9*), 1868–1872	7/33	21.2 (7.3–35.1)	16/33	48.5 (31.5–65.4)	8/10	80.0 (55.3–100.0)
Welch (*10*), 1878	8/12	66.7 (40.1–93.2)	9/22	40.9 (20.5–61.3)	10/12	83.3 (62.4–100.0)
Queirel (*18*), 1906	3/4	75.0 (32.8–100.0)	8/10	80.0 (55.3–100.0)	0/5	0 (NC)
Robertson (*19*), 1913	1/2	50.0 (0.0–100.0)	6/9	66.7 (36.0–97.3)	1/12	8.3 (0.0–23.9)
Rao (*5*), 1959–1962	10/21	47.6 (26.4–68.9)	16/65	24.6 (14.2–35.0)	41/94	43.6 (33.6–53.6)
Total	29/72	40.3 (29.0–51.5)	55/139	39.6 (31.5–47.7)	60/133	45.1 (36.7–53.5)

*L/C, miscarriage or premature birth/cases; PL, proportion of miscarriage and premature birth; CI, confidence interval; NC, not calculable.

These outcomes could only be compared by history of miscarriage in the 1913 outbreak in Australia (*19*). Two of 3 patients with no history and 6 of 20 with a history of miscarriage had a miscarriage or premature birth, but this difference was not significant (p = 0.27, odds ratio 4.7, 95% CI 0.4–61.8). Comparison by previous experience of normal delivery (primipara or multipara) could only be performed with the data from Rotterdam from 1893 and 1894 (*15*). Ten of 21 primipara patients and 18 of 53 multipara patients had a miscarriage or premature birth (p = 0.30), which suggests that delivery history did not greatly affect the outcome of pregnancy complicated by smallpox.

## Conclusions

Since outbreaks have been limited since the mid-20th century by the successful smallpox eradication program, historical records are a useful tool to document common patterns of maternal outcomes in pregnancy complicated by smallpox. Such analysis may be limited by unknown numbers of missed or unreported cases or imperfect vaccination histories. My estimates of the overall crude case fatality and proportion of miscarriage or premature birth were high. This study and Rao's (*4*) improve our understanding of smallpox in pregnancy, highlighting 3 points. First, case fatality is highest during the last trimester of gestation, but miscarriage and premature birth do not vary by trimester. Physiologic changes in the third trimester could partly explain the higher case fatality (*21*). Second, even mild cases were at high risk of causing miscarriage or premature birth. Third, miscarriage and premature birth were not significantly associated with vaccination history or previous miscarriage or delivery. That is, vaccination may not prevent miscarriage and premature birth.

Although prior vaccination offers less protection to pregnant women than others (*22*), this study shows that vaccination might offer at least partial protection. Case fatality in the event of a bioterrorist attack could be lowered with vaccination before pregnancy and should be considered if the risk for such an attack is high.

## Appendix 1

### Methods

#### Search Strategy

**Figure A1:**
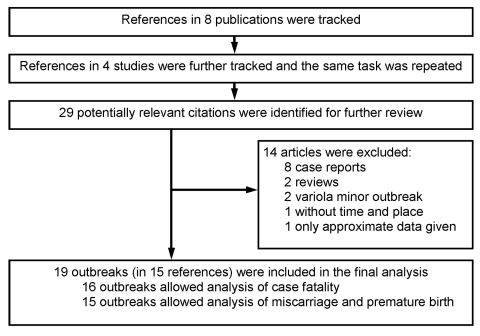
Flow diagram of the study selection process.

Since most documented large-scale outbreaks of smallpox were written before the mid-20th century, this study could not follow formal methods of systematic review, i.e., by using MEDLINE or other databases. Consequently, this study first tracked selected references in major specialized books (1–4) and articles on smallpox or viral diseases after the mid-20th century (5–8). These references (9–12) were subsequently reviewed, and their references were further tracked, regardless of language, to search for potentially useful articles. This task was repeated as many times as necessary until no further references were identified (Figure A1). Consequently, references dating back to the 19th century were reviewed.

#### Study Selection Criteria

All publications reporting either of the defined maternal outcomes, i.e., case fatality and miscarriage or premature birth, were selected. Second, the obtained publications were limited by the following inclusion criteria: the publication must 1) document >8 smallpox cases during pregnancy (exclusion of case reports), 2) clarify the time and place of the outbreak, 3) report an outbreak of variola major (not variola minor), and 4) explicitly define and describe either of the outcomes as a major topic of discussion.

### Statistical Analysis

Since crude case fatalities and frequencies of miscarriage and premature birth could be biased by several serious underlying factors, e.g., vaccination and progress in obstetrics and medicine as a whole, the heterogeneity of which cannot be methodologically adjusted, interpreting findings after combining the data after statistical adjustment was deemed inappropriate in this study. Thus, data analysis did not follow usual methods of metaanalysis for estimation of combined case fatality and the proportion of miscarriage and premature birth. Rather, the outcomes, except overall crude estimates, were investigated by each publication separately when possible. The χ^2^ and Fisher exact tests were used to evaluate univariate associations. Confidence intervals (CI) for a proportion, *p*, were obtained by using the standard error,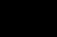, where N is the sample size.

### Identification of Historical Records

First, 13 studies were excluded: 8 were clinical case reports (13–20), 1 did not clarify the time and place of the outbreak and showed the same number of cases and deaths documented in another study included for analysis (21), 2 documented variola minor outbreaks (6,9), and the remaining 2 were review articles that did not provide outcomes (22,23). Further, a more recent study by Dixon in Tripolitania was also excluded because only approximate case fatalities (40% and 11% for pregnant and nonpregnant patients, respectively) were given (*24*). Four patients in the New South Wales report (*25*) were excluded from the analyses because disease onset was observed after delivery. Consequently, as noted in the main text, 19 outbreaks were identified. Half originated from a review article published in 1932 (10). The original reports were written in Dutch, English, French, German, and Italian.

### Appendix 1 References

1. Fenner F, Henderson DA, Arita I, Ježek Z, Ladnyi ID. Smallpox and its eradication. Geneva: World Health Organization. 1988 [cited 2006 May 3]. Available from http://whqlibdoc.who.int/smallpox/9241561106.pdf
2. Dixon CW. Smallpox. London: Churchill; 1962
3. Rao AR. Smallpox. Bombay: Kothari Book Dept; 1972.
4. Hanshaw JB, Dudgeon JA. Viral diseases of the fetus and newborn. Philadelphia: Saunders; 1978.
5. Rao AR, Prahlad I, Swaminathan M, Lakshmi A. Pregnancy and smallpox. J Indian Med Assoc. 1963;40:353–63. PubMed
6. Megale P, Angulo JJ, Pederneiras CA. Variola minor in Braganca Paulista county, 1956. Observations on the clinical course of variola minor and on pregnancy in women with the disease. Bull Soc Pathol Exot. 1979;72:11–20. PubMed
7. Marsden JP. Variola minor; a personal analysis of 13,686 cases. Bull Hyg (Lond). 1948;23:735–46.
8. Saxen L, Cantell K, Hakama M. Relation between smallpox vaccination and outcome of pregnancy. Am J Public Health Nations Health. 1968;58:1910–21.
9. Marsden JP, Greenfield CRM. Inherited smallpox. Arch Dis Child. 1934;9:309–14.
10. Lynch FW. Dermatologic conditions of the fetus with particular reference to variola and vaccinia. Arch Derm Syphilol. 1932;26:997–1019.
11. Bancroft IR. Clinical observations on variola. Journal of Medical Research. 1904;11:322–44.
12. Voigt L. Über den Einfluss der Pockenkrankheit auf Menstruation, Schwangerschaft, Geburt und Fötus. Sammlung Klinischer Vortraege/Gynaekologie. 1894–1897;112:249–72.
13. Lop PA. Variole et vaccine dans la grossesse [thesis]. Paris: Université de Paris; 1893.
14. Jenner E. Two cases of Small-pox infection communicated to the foetus in utero under particular circumstances, with additional remarks. Medico-Chirurgical Transactions. 1809;1:269–75.
15. Davidson W. Small-pox in utero. Lancet. 1837–1838;2:628.
16. Marsden JP, Chir B. Metastatic calcification. Notes on twins born shortly after an attack of smallpox in the mother. Br J Child Dis. 1930;27:193–200.
17. Rigden G. Influence of maternal small-pox on the fetus. BMJ. 1877;1:229–30.
18. Robinson H. Occurrence of confluent small-pox at the seventh month of pregnancy. BMJ. 1877;1:163.
19. Cowie JM, Forbes D. Intrauterine influence of the fetus in small-pox. BMJ. 1904;1:1485.
20. Paranjothy D, Samuel I. Pregnancy associated with haemorrhagic smallpox. J Obstet Gynaecol Br Emp. 1960;67:309–13. PubMed
21. Pfeiffer L. Behandlung und Prophylaxe der Blattern. Handbuch der speciellen Therapie. Jena (Germany): Fischer; 1894.
22. von Jarrier. De la menstruation dans la variole [thesis]. Paris: Université de Paris; 1880.
23. von Obermeier. Beiträge zur Kenntnis der Pocken. Virchows Arch. 1872;IV:S345.
24. Dixon CW. Smallpox in Tripolitania, 1946: an epidemiological and clinical study of 500 cases, including trials of penicillin treatment. J Hyg (Lond). 1948;46:351–77. PubMed
25. Robertson DG. Small-pox epidemic in New South Wales, 1913. Melbourne, Australia: Minister for Trade and Customs; 1914.

## Appendix 2

### Additional Data and Discussion

#### Additional Data and Results

Appendix 2 Table A1 and Table A2 stratify case fatalities and the proportion of miscarriage or premature birth by the clinical classification of smallpox. Appendix 2 Table A3 and Table A4 compare the frequency of death among pregnant and nonpregnant patients and stratify the frequency by vaccination history.

Appendix 2 Table A5 shows the frequency of miscarriage or premature birth by clinical stage of smallpox among 27 patient in Philadelphia in 1878 (9); all patients miscarried or delivered prematurely at the given date of the illness. Fourteen patients (51.9%) miscarried or delivered prematurely within 5 days after rash appeared, while the frequency among the remainder showed a long-tailed distribution. Appendix 2 Table A6, Table A7, Table A8 provide anonymous individual records of 46, 19, and 23 pregnant smallpox patients in Philadelphia (10), Paris (5), and New South Wales (7), respectively. The investigated variables differed by outbreak.

### Supplementary Discussion: Validity and Reliability

A few specific limitations of this study must be addressed. The first is related to the underdiagnosis of pregnancy, especially in the early gestational period. Moreover, the definition of pregnancy-related deaths is difficult to grasp, even at present (11). Whereas this limitation could have led to overestimation of miscarriage, case fatality is not thought to have been substantially influenced, especially since the sample size was large. Second, regarding the reliability of the data, some of the earliest epidemiologic studies were performed before maturation of both the epidemiologic and statistical methods used in current epidemiologic observations. For example, technical problems arose when precise epidemiologic interpretation was needed: 1) adjusting confounding variables was extremely difficult, and I refrained from further stratifying for adjustment or multivariate analysis with the limited number of cases, and 2) the cases classified as variola sine eruptione shown here did not follow virologic confirmation, and diagnosis of this type was made mainly on the basis of probable contacts. Nevertheless, other types of variola major can be confidently diagnosed compared to other infectious diseases, and historical records remain a useful tool as long as the literature appropriately documents the necessary data. This study was motivated by the relatively high reliability of diagnosis and determination of both fatality and miscarriage or premature birth, obvious events compared to fetal vaccinia, which is extremely difficult to diagnose, and fetal and neonatal outcomes, which could be biased by progress in obstetrics and medicine on a whole. Although adhering to formal methods of metaanalysis and showing combined estimates of maternal outcomes with adjustment was difficult, this study successfully confirmed that smallpox is more severe with pregnancy and characterized several features of maternal outcomes.

### Appendix 2 References

1. Meyer L. Über Pocken beim weiblichen Geschlecht. Beiträge zur Geburtshülfe und Gynäkologie / hrsg. von d. Gesellschaft für Geburtshülfe in Berlin (Berlin: Crede). 1873;2:186–98.
2. Sangregorio G. Vaiuolo e gravidanza. Cenni statistici (1). Guardia Ostetrica di Milano. I Morgagni. 1887;29:793–6.
3. van der Willigen AM. Pokken in de Zwangerschap, 80 gevallen van variolae gravidarum. Ned Tijdschr Geneeskd. 1895;11:485–99.
4. Charpentier JB. Variole et vaccine dans la grossesse [thesis]. Paris: Université de Paris; 1900.
5. Queirel. Variole et grossesse. Annales de Gynecologie et d'Obstetrique. 1907;4:137–47.
6. Rao AR. Haemorrhagic smallpox: a study of 240 cases. J Indian Med Assoc. 1964;43:224–9.
7. Robertson DG. Small-pox epidemic in New South Wales, 1913. Melbourne: issued under the authority of the Minister for Trade and Customs; 1914.
8. Rao AR. Smallpox. Bombay: Kothari Book Dept; 1972.
9. Fenner F, Henderson DA, Arita I, Ladnyi ID. Smallpox and its eradication. Geneva: World Health Organization; 1988 [cited 2006 May 4]. Available from http://whqlibdoc.who.int/smallpox/9241561106.pdf
10. Welch WM. Smallpox in the pregnant woman and in the foetus. Philadelphia Medical Times. 1877–1878;8:390–8.
11. Deneux-Tharaux C, Berg C, Bouvier-Colle MH, Gissler M, Harper M, Nannini A, et al. Underreporting of pregnancy-related mortality in the United States and Europe. Obstet Gynecol. 2005;106:684–92.

## Figures and Tables

**Figure:**
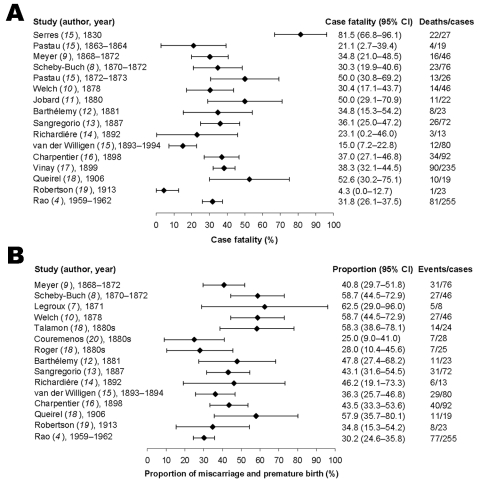
Maternal outcomes in pregnancies complicated by smallpox from data from 19th- and early 20th-century outbreaks. A) Case fatalities and B) proportions of miscarriage or premature birth are shown. Miscarriage and premature birth before maternal death are included. For additional study information, see references.

## Appendix

**Table A1:** Case fatality among pregnant women with smallpox by clinical types of variola major, according to data from 19th- and early 20th-century outbreaks*

Reference	Hemorrhagic		Confluent		Discrete		VSE	
	D/C	CF (95% CI)	D/C	CF (95% CI)	D/C	CF (95% CI)	D/C	CF (95% CI)
Meyer (1), 1868–1872	13/13	100.0 (NC)	9/26	34.6 (16.3–52.9)	–	–	0/37	0.0 (NC)
Sangregorio (2), 1887	3/3	100.0 (NC)	20/22	90.9 (78.9–100.0)	3/40	7.5 (0.0–15.7)	0/7	0.0 (NC)
van der Willigen (3), 1893–1894	6/6	100.0 (NC)	4/4	100.0 (NC)	2/10	20.0 (0.0–44.8)	0/60	0.0 (NC)
Charpentier (4), 1898	13/13	100.0 (NC)	17/34	50.0 (33.2–66.80	4/45	8.9 (0.6–17.2)	–	–
Queirel (5), 1906	8/8	100.0 (NC)	2/3	66.7 (13.3–100.0)	0/8	0.0 (NC)	–	–
Rao (6), 1959–1962	14/14	100.0 (NC)	12/32	37.5 (20.7–54.3)	0/48	0.0 (NC)	–	–

*Hemorrhagic, widespread hemorrhages in the skin and mucous membranes; confluent, confluent rash on the face and arms; discrete, areas of normal skin visible between pustules, even on the face; VSE, variola sine eruptione, fever without rash caused by variola virus, also known as varioloid (*8*,*9*); D/C, Smallpox deaths/cases; CF, case fatality; CI, confidence interval; NC, not calculable.

**Table A2:** Miscarriage or premature birth among pregnant women with smallpox by clinical types of variola major, according to data from 19th- and early 20th-century outbreaks

Reference	Hemorrhagic		Confluent		Discrete		VSE	
	L/C	PL (95% CI)	L/C	PL (95% CI)	L/C	PL (95% CI)	L/C	PL (95% CI)
Meyer (1), 1868–1872	13/13	100.0 (NC)	14/26	53.8 (34.7–73.0)	–	–	4/37	10.8 (0.8–20.8)
Sangregorio (2), 1887	3/3	100.0 (NC)	17/22	77.3 (59.8–94.8)	10/40	25.0 (11.6–38.4)	1/7	14.3 (0.0–40.2)
Charpentier (4), 1898	13/13	100.0 (NC)	18/34	52.9 (36.2–69.7)	9/45	20.0 (8.3–31.7)	–	–
Queirel (5), 1906	8/8	100.0 (NC)	3/3	100.0 (NC)	0/8	0.0 (NC)	–	–

*Hemorrhagic, widespread hemorrhages in the skin and mucous membranes; confluent, confluent rash on the face and arms; discrete, areas of normal skin visible between pustules, even on the face; VSE, variola sine eruptione, fever without rash caused by variola virus, also known as varioloid (*8*,*9*); L/C, miscarriage or premature birth/cases; PL, proportion of miscarriage or premature birth; CI, confidence interval; NC, not calculable.

**Table A3:** Comparison of the frequency of deaths among pregnant and nonpregnant patients, according to data from 19th- and early 20th-century outbreaks*

Reference	Nonpregnant		Pregnant			
	Cases	Deaths	Cases	Deaths	p value*	OR (95% CI)†
Meyer (1), 1868–1872	1116	163	76	23	<0.01	2.5 (1.5–4.3)
van der Willigen (3), 1893–1894	352	39	80	12	0.33	1.4 (0.7–2.8)
Rao (6), 1959–1962	348	29	94	26	<0.01	4.2 (2.3–7.6)

*2-sided. †OR, odds ratio; CI, confidence interval.

**Table A4:** Comparison of the frequency of deaths stratified by vaccination history, according to data from 19th- and early 20th-century outbreaks

Reference	Unvaccinated		Vaccinated			
	Cases	Deaths	Cases	Deaths	p value*	OR (95% CI)†
Welch (10), 1878	7	7	39	7	<0.01	NC
van der Willigen (3), 1893–1894	2	2	78	10	0.02	NC
Rao (6), 1959–1962	12	9	82	17	<0.01	11.5 (2.8–47.1)

*2-sided. †OR, odds ratio; CI, confidence interval; NC, not calculable.

**Table A5:** Frequency of miscarriage or premature birth with smallpox by clinical stage of symptoms, Philadelphia, 1878 (10)

Stage of illness	n
Prodromal period	1
Eruption day 1	4
Day 2	2
Day 3	3
Day 4	2
Day 5	2
Days 6–10	1
Days 11–20	1
Days 21–30	3
Day 31 onwards	3
No precise description	5
Total	27

**Table A6:** Anonymous records of 46 pregnant women with smallpox, Philadelphia, 1878 (10)

Patient identification	Age	Classification*	Vaccination history	Gestational age (mo)	Dates of smallpox at miscarriage or premature birth†	Maternal outcome
1	23	Variola	Vaccinated	4	Day 4 of eruption	Recovered
2	27	Variola	Vaccinated	3		Died
3	35	Varioloid	Vaccinated	3	Day 18 of eruption	Recovered
4	21	Variola	Vaccinated	2	Week 3	Recovered
5	32	Varioloid	Vaccinated	5	After discharge	Recovered
6	24	Variola	Vaccinated	8	Day 1 of fever	Died
7	30	Variola	Vaccinated	5.5		Recovered
8	26	Varioloid	Vaccinated	3	5 wks after discharge	Recovered
9	22	Variola	Unvaccinated	7.5	Day 1 of eruption	Died
10	35	Varioloid	Vaccinated	3		Recovered
11	15	Variola	Unvaccinated	3		Died
12	23	Varioloid	Vaccinated	2	Day 10 after discharge	Recovered
13	22	Varioloid	Vaccinated	5.5	Day 26 of eruption	Recovered
14	18	Varioloid	Vaccinated	8.5	Day 9 of eruption	Recovered
15	21	Varioloid	Vaccinated	3		Recovered
16	29	Varioloid	Vaccinated	5		Died
17	30	Varioloid	Vaccinated	9	Early stage	Recovered
18	30	Variola	Vaccinated	5.5	Day 2 of eruption	Died
19	27	Varioloid	Vaccinated	7		Recovered
20	27	Variola	Vaccinated	3	Day 1 of eruption	Died
21	26	Variola	Unvaccinated	7 or 8	Day 3 of eruption	Died
22	32	Varioloid	Vaccinated	8		Recovered
23	20	Variola	Unvaccinated	6		Died
24	17	Variola	Unvaccinated	4	6 wks after discharge	Recovered
25	24	Variola	Vaccinated	3		Died
26	19	Varioloid	Vaccinated	6	1 mo after discharge	Recovered
27	26	Variola	Vaccinated	5	Day 3 of eruption	Died
28	22	Varioloid	Vaccinated	3	Day 2 of eruption	Recovered
29	30	Variola	Vaccinated	5.5	Day 4 of eruption	Recovered
30	20	Varioloid	Vaccinated	8	Day 1 of eruption	Recovered
31	25	Variola	Vaccinated	5.5		Recovered
32	25	Variola	Vaccinated	4	Day 5 of eruption	Recovered
33	45	Varioloid	Vaccinated	6		Recovered
34	19	Varioloid	Vaccinated	6		Recovered
35	26	Variola	Unvaccinated	7.5	Day 1 of eruption	Died
36	18	Varioloid	Vaccinated	3.5		Recovered
37	26	Variola	Unvaccinated	8	Early stage	Died
38	41	Varioloid	Vaccinated	8	Day 3 of eruption	Recovered
39	28	Varioloid	Vaccinated	4.5		Recovered
40	30	Varioloid	Vaccinated	6		Recovered
41	25	Varioloid	Vaccinated	3	Day 1 of eruption	Recovered
42	20	Variola	Vaccinated	6.5	Day 5 of eruption	Died
43	22	Varioloid	Vaccinated	5		Recovered
44	28	Varioloid	Vaccinated	6		Recovered
45	21	Varioloid	Vaccinated	5	During maturation	Recovered
46	25	Varioloid	Vaccinated	6		Recovered

*Variola includes a rash (hemorrhagic, confluent, and discrete), while varioloid is equivalent to variola sine eruptione. †Clinical stage of smallpox when miscarriage or premature birth occurred. Those reports that did not document the outcomes have been left blank.

**Table A7:** Anonymous records of 19 pregnant women with smallpox, Paris, 1906 (5)

Patient identification	Gestational age (mo)	Miscarriage or premature birth*	Maternal outcome
1	3	Yes	Died
2	4	Yes	Died
3	5	Yes	Died
4	5	Yes	Died
5	5	Yes	Died
6	6	Yes	Died
7	6	Yes	Died
8	7	No	Died
9	2	Yes	Recovered
10	2.5	No	Recovered
11	3	Yes	Died
12	4	Yes	Died
13	5	No	Recovered
14	6	Yes	Recovered
15	6.5	No	Recovered
16	7	No	Recovered
17	8	No	Recovered
18	9	No	Recovered
19	9	No	Recovered

*Distinction between miscarriage and premature birth was not made.

**Table:** Table A8. Anonymous records of 23 pregnant women with smallpox, New South Wales, 1913 (7)

Patient identification	Age (y)	Previous miscarriage?	Gestational age (mo)	Miscarriage or premature birth?
1	20	No	3	Yes
2	27	Yes	6	Yes
3	19	No	4	Yes
4	34	No	4	Yes
5	20	No	7.5	No
6	29	No	8	No
7	35	No	5	No
8	25	No	7	No
9	24	Yes	7	No
10	25	No	7	No
11	22	No	8	No
12	26	No	8	No
13	28	No	5	No
14	30	No	Late stage	Yes
15	28	No	6	No
16	21	No	8	No
17	32	No	3.5	Yes
18	26	No	2	No
19	38	No	7	No
20	24	No	7	No
21	24	No	3.5	Yes
22	20	No	8	0
23	27	Yes	6	Yes
